# Nodular Lymphangitis Syndrome

**DOI:** 10.4269/ajtmh.17-0397

**Published:** 2017-11-08

**Authors:** Álvaro A. Faccini-Martínez, Raphael L. Zanotti, Maithê S. Moraes, Aloísio Falqueto

**Affiliations:** 1 Postgraduate Program in Infectious Diseases, Universidade Federal do Espírito Santo, Vitória, ES, Brazil;; 2 Health Science Center, Universidade Federal do Espírito Santo, Vitória, ES, Brazil;; 3 School of Medicine, Universidade Federal do Espírito Santo, Vitória, ES, Brazil

## INTRODUCTION

Between August 2016 and April 2017, three patients from different towns in the state of Espírito Santo, Brazil, presented with nodular lymphangitis with diverse presentations.

## CASE 1

**Figure 1.**
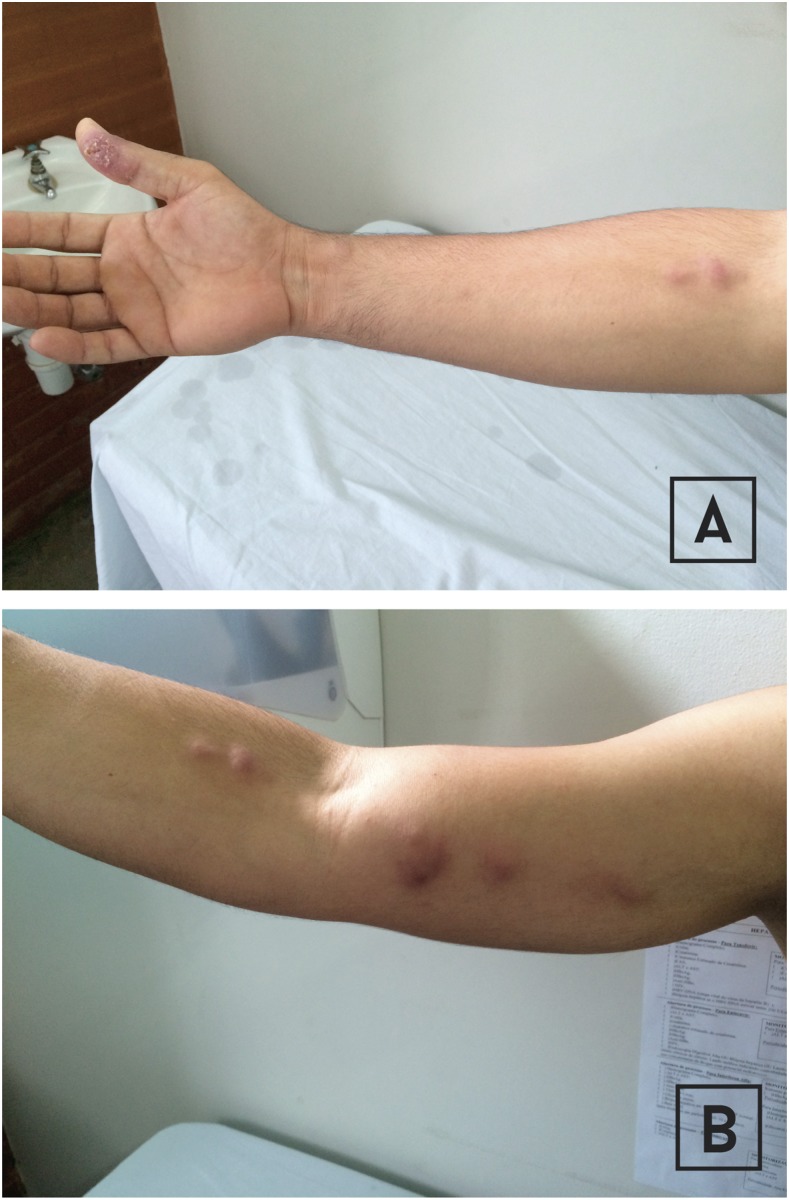
(**A**) Ulcerated lesion on right thumb and subcutaneous erythematous nodules along lymphangitic streak in the forearm. (**B**) Subcutaneous erythematous nodules along lymphangitic streak in the arm and forearm. This figure appears in color at www.ajtmh.org.

On August 19, 2016, a 36-year-old man from an urban area of Cariacica town, an electricity worker, presented with refractory cellulitis involving his right thumb and extending up his arm. Two months before, he had pricked his thumb with a wood splinter. Within 10 days, a painless papule appeared at the inoculation site, and over the next weeks, tender lymphangitis developed. Despite the use of oral cephalexin and amoxicillin-clavulanate, lymphangitis involving the forearm progressed. He was afebrile, and there were no systemic symptoms or signs. An ulcerated lesion on the affected finger and subcutaneous erythematous nodules in a lymphangitic streak were observed (Figure 1). Right axillary lymphadenitis was noted. Taking into account the epidemiological exposure history, nodular lymphangitis by *Nocardia* spp. was suspected and oral therapy with trimethoprim-sulfamethoxazole was indicated (160–800 mg po twice a day) for a total of 3 months, with complete resolution of lesions.

## CASE 2

**Figure 2.**
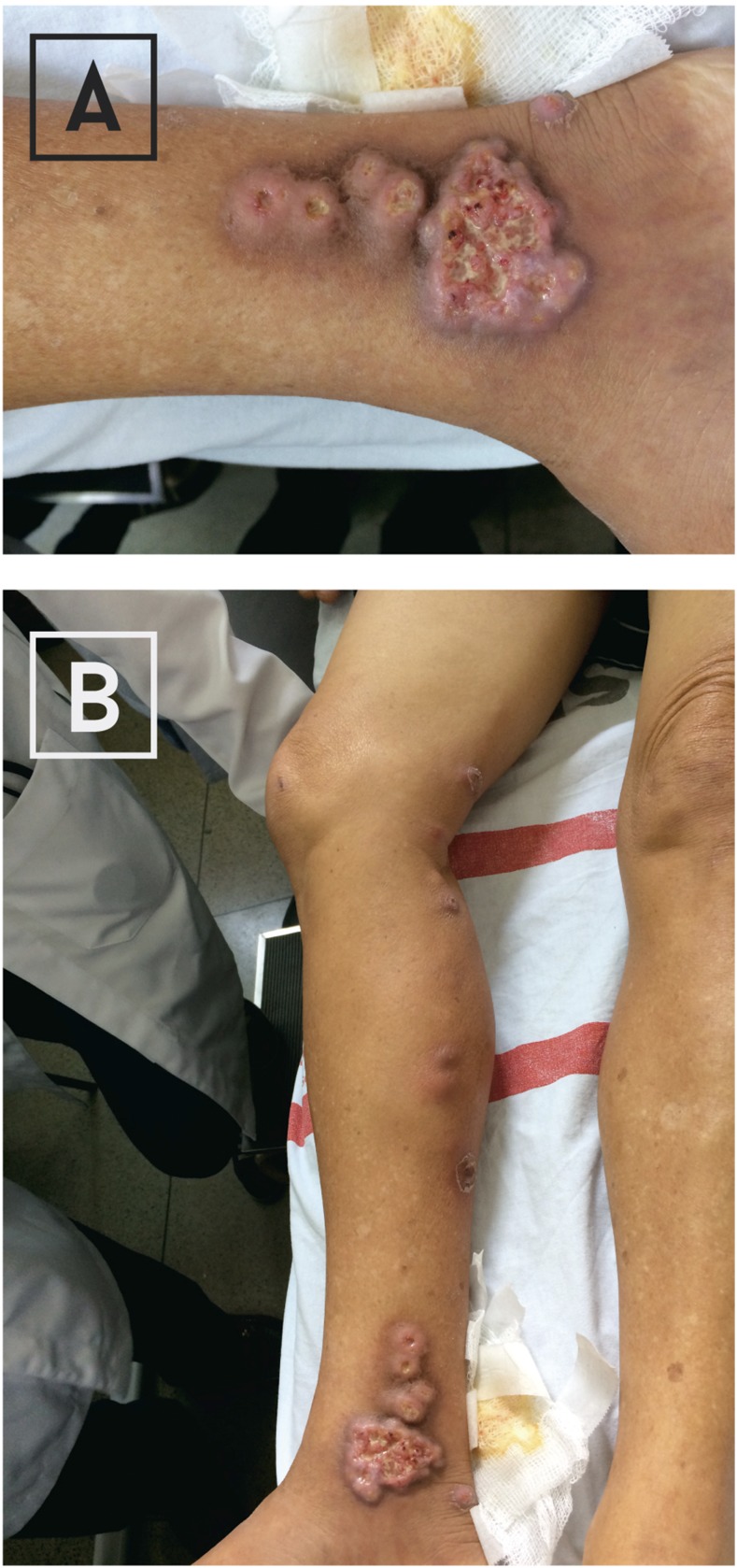
(**A**) Ulcers with irregular borders and satellite microabscesses on the right ankle. (**B**) Ulcers with irregular borders and satellite microabscesses on the right ankle and subcutaneous erythematous nodules along lymphangitic streak in the involved leg. This figure appears in color at www.ajtmh.org.

On October 25, 2016, a 56-year-old woman from the rural town of Iconha, farmer, had a 3-month history of ulcerated lesions on the inner right ankle with tender ipsilateral lymphangitis. The ankle lesions started as a single erythematous papule, which progressively grew, became ulcerated and was associated with satellite lesions. Despite the use of oral amoxicillin-clavulanate, lymphangitis involving the leg progressed. She was afebrile, and there were no systemic symptoms or signs. Enlarged lymph nodes were not present. The patient denied contact with animals or recent local trauma. Ulcers with irregular borders and satellite microabscesses on the affected ankle and subcutaneous erythematous nodules along lymphangitic streak were seen on the involved leg (Figure 2). Samples obtained from the ulcerated lesions were inoculated on Sabouraud dextrose agar and incubated at 25°C. *Sporothrix* sp. was isolated, as assessed by the growth of typically filamentous colonies. Patient was treated with a saturated solution of potassium iodide administered orally, twice daily, at a daily oral dose of 50 mg iodine/drop/kg, for a total of 40 days. Lesions resolved.

## CASE 3

**Figure 3.**
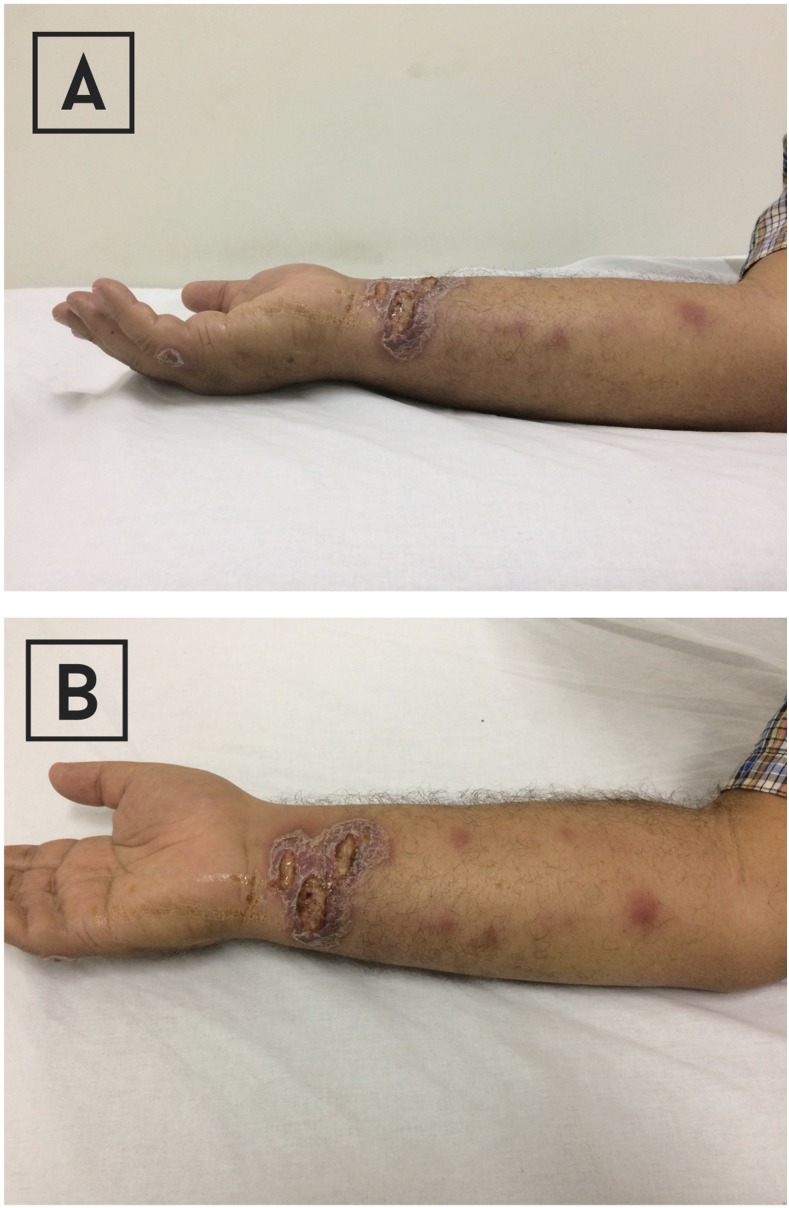
(**A**) Confluent ulcers with an indurated raised outer border on the right wrist, ipsilateral satellite lesion in the little finger, and subcutaneous erythematous nodules in the forearm (lateral view). (**B**) Confluent ulcers with an indurated raised outer border on the right wrist, ipsilateral satellite lesion in the little finger, and subcutaneous erythematous nodules along two lymphangitic streaks in the involved arm. This figure appears in color at www.ajtmh.org.

On April 17, 2017, a 62-year-old man from a rural area of Ibatiba town, farmer, with history of type two diabetes mellitus, presented with 4-month history of ulcerated lesions on the inner right wrist with tender ipsilateral lymphangitis. The wrist lesions started as progressively expanding erythematous papules in the forearm that progressed to ulceration, and formation of nodules. He was afebrile, and there were no systemic symptoms or signs. Enlarged lymph nodes were not observed. There was no history of local trauma. Confluent ulcers with an indurated raised outer border on the affected wrist, ipsilateral satellite lesion in the little finger and subcutaneous erythematous nodules along two lymphangitic streaks were observed (Figure 3A and B). A diagnosis of nodular lymphangitis due to *Leishmania* spp. was established with the finding of amastigotes on scrapings from the border of the ulcers and intravenous therapy with liposomal amphotericin B was indicated (1.5 mg/kg/daily) for a total of 18 days, with complete resolution of lesions.

Nodular lymphangitis syndrome is characterized by the development of superficial cutaneous lesions that progress with nodules along dermal and subcutaneous lymphatics.^1^ The classic clinical pattern is described as “sporotrichoid” after the infection caused by the dimorphic fungus *Sporothrix schenckii*. This syndrome may be produced by fungal, bacterial, mycobacterial, parasitic, and viral pathogens that are environmental inhabitants of soil and/or vegetative matter, and traumatic inoculation from a source in nature initiates the process of infection.^1,2^ Nodular lymphangitis develops most commonly after cutaneous inoculation with *S. schenckii*, *Nocardia brasiliensis*, *Leishmania* (*Viannia*) *braziliensis*, and *Mycobacterium marinum*.^1–4^ A detailed history, accompanied by information obtained from skin biopsy specimens with appropriate stains and cultures, should allow specific, affective therapy for most of these infections.^4^
